# Evaluating contralateral neck failure in patients with lateralized OPSCC treated with transoral robotic surgery and neck management based on pre-operative SPECT-CT lymphatic mapping

**DOI:** 10.1186/s40463-022-00563-z

**Published:** 2022-12-27

**Authors:** Ilyes Berania, Ali Hosni, Carissa M. Thomas, David Goldstein, Andrew Bayley, Ravi Mohan, Aaron Hendler, Richard M. Cooper, John R. de Almeida

**Affiliations:** 1 Department of Otolaryngology—Head and Neck Surgery, Princess Margaret Cancer Centre, University Health Network, University of Toronto, 610 University Avenue, Suite 3-950, Toronto, ON M5G 2M9 Canada; 2 Department of Surgical Oncology, Princess Margaret Cancer Centre, University Health Network, Toronto, ON Canada; 3 Department of Radiation Oncology, University of Toronto, Toronto, ON Canada; 4 Department of Nuclear Medicine, Joint Department of Medical Imaging, University Health Network, Toronto, ON Canada; 5 Department of Anesthesia, University of Toronto, Toronto, ON Canada

**Keywords:** Lymphoscintigraphy, Neck dissection, Oropharyngeal squamous cell carcinoma, Sentinel lymph node biopsy, SPECT-CT, Transoral robotic surgery

## Abstract

**Background:**

Risk of contralateral nodal metastases in oropharyngeal squamous cell carcinoma (OPSCC) is relatively low, however, many OPSCC patients receive bilateral neck treatment. This study evaluates the oncological outcomes with management of the contralateral cN0 neck based on lymphatic mapping with single photon emission computed tomography (SPECT-CT).

**Methods:**

Retrospective evaluation of patients with lateralized cT1-2 and contralateral cN0 OPSCC treated with primary surgery between December 2017 and October 2019. All patients underwent pre-operative lymphatic mapping using SPECT-CT. Clinical parameters including demographics, tumor characteristics and oncological outcomes were recorded.

**Results:**

Thirteen patients underwent primary site resection with transoral robotic surgery (TORS) and ipsilateral neck dissection with or without adjuvant therapy. Twelve patients (92.3%) had ipsilateral drainage on SPECT-CT, whereas 1 (7.7%) patient had bilateral neck lymphatic drainage. Four patients (30.8%) underwent post-operative radiation therapy (PORT). Three patients with unilateral drainage on SPECT-CT underwent PORT with unilateral neck irradiation, and 1 patient with bilateral drainage underwent PORT with bilateral neck irradiation. Seven (53.8%) patients were staged as pT1, 6 (46.2%) patients as pT2, 6 (46.2%) patients were pN0, 3 (23.1%) patients were pN1, 1 (7.7%) patient was pN2a for and 3 (23.1%) patients were N2b. The median distance of the tumor from midline was 1.05 cm (0.0–1.58). Primary sites included tonsil (n = 10, 76.9%) and tongue base (n = 3, 23.1%). The median follow-up time was 15.4 months. All patients were disease free at the latest follow-up with no contralateral neck failures.

**Conclusions:**

Pre-operative mapping of lymphatic drainage in early stage OPSCC with SPECT-CT is a promising tool which can reduce treatment to the contralateral neck potentially without compromising oncological outcomes.

**Graphical Abstract:**

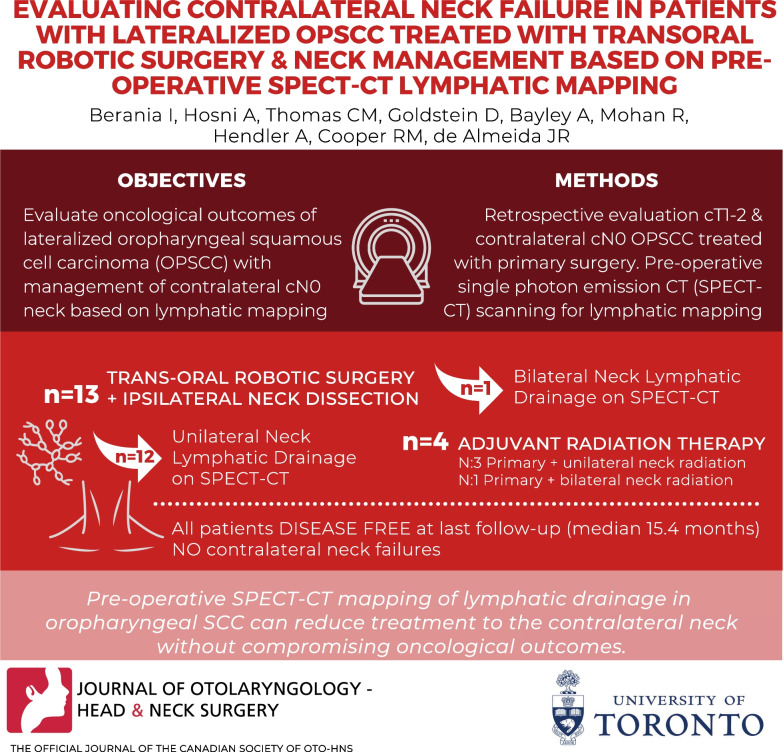

## Introduction

The incidence of oropharyngeal squamous cell carcinoma (OPSCC) has been steadily increasing and is expected to continue to rise over the next decades [1]. Research efforts are currently focused on understanding oropharyngeal tumor biology and patterns of spread, including pathways of lymphatic invasion [2]. Nodal status has a critical importance on prognosis and therapeutic planning for oropharyngeal cancer [3]. OPSCC has a propensity to spreads through lymphatic channels, with a significant rate of occult nodal metastasis requiring primary management of the neck [4]. Appropriate treatment of the contralateral cN0 neck for early stage OPSCC remains under investigation. Several studies suggest that early stage and well lateralized tumors of the oropharynx have predictable lymphatic drainage limited to the ipsilateral neck, although most institutions currently manage OPSCC patients with bilateral neck treatment [5]. Multiples reports have demonstrated that bilateral neck treatment for lateralized tumors of the oropharynx with a cN0 contralateral neck is associated with a low rate of occult contralateral neck disease, estimated to be less than 10% [6–8].

While the risk of contralateral nodal spread is low, additional studies are required to assess the oncological safety of de-escalation protocols avoiding treatment to the contralateral neck. Although several studies previously reported a low incidence of contralateral nodal metastasis [7, 8], these results should be interpreted with caution due to the variability of HPV related oropharyngeal cancer, inclusion of advanced stage tumors, and clinical rather than pathological estimation of nodal status using non-surgical therapies. Oropharyngeal malignancies can have variable lymphatic drainage patterns based on their anatomical location. Tumors close to the midline, such soft palate and tongue base cancers, are higher risk for bilateral lymphatic drainage and require bilateral neck treatment [9].

The introduction of novel, high-resolution imaging tools such as SPECT-CT lymphoscintigraphy into the pre-operative evaluation of OPSCC has allowed for the accurate assessment of lymphatic drainage patterns in the neck. Pre-operative lymphatic mapping is a well-tolerated, minimally invasive procedure that can be performed on awake patients. This method is a reliable tool, characterizing lymphatic spread in 90% of tumors [10]. Identifying tumor-specific lymphatic drainage patterns allows for tailored treatments to nodal basins at greatest risk for regional disease spread. Avoiding treatment of the contralateral neck could significantly reduce treatment related toxicities and improve patient’s quality of life [11]. The present study evaluates the oncological outcomes and incidence of early contralateral neck failure among patients with early stage OPSCC who underwent primary surgical management, including neck treatment based on pre-operative lymphatic mapping with SPECT-CT.

## Methods

Institutional Research Ethics Board (19-5071) was obtained for the study. We retrospectively assessed patients (n = 13) treated surgically for early stage (T1–T2) OPSCC without clinical evidence of contralateral nodal involvement from December 2017 through October 2019. All patients underwent pre-operative lymphatic mapping using SPECT-CT followed by primary transoral robotic surgery (TORS) and ipsilateral neck dissection. Inclusion criteria was lateralized tumors not crossing midline, without evidence of contralateral nodal disease on axial imaging (either CT and/or MRI). Adjuvant radiation therapy was delivered to the primary site and neck based on standard indications such as close (< 5 mm) or positive resection margins, the presence of multiple positive nodes (≥ 2) or any node(s) > 3 cm, the presence of perineural invasion or lymphovascular invasion, and/or the presence of extranodal extension. Contralateral neck adjuvant radiotherapy was only delivered in cases where the lymphatic map labelled contralateral lymphatic drainage. Clinical parameters including patient demographics, tumor characteristics, final pathology results and oncological outcomes were recorded. The AJCC, 7th edition was used for TNM tumor staging. Tumor distance to midline was measured from the preoperative Magnetic Resonance Imaging (MRI), T2 sequence. The distance was measured from the most medial point of the tumor to the midline (cm). The midline was defined as a sagittal plane from the midpoint of the cricoid cartilage.

### Tumor injection for lymphatic mapping

A detailed description of the injection technique was previously described by our group in an initial feasibly study [10]. All radiotracer injections were performed awake in the Nuclear Medicine Suite. All injections procedures were performed by a single surgeon (J.D.A.) with the initial collaboration of an anesthesiologist (RMC). The injection and imaging were performed the day prior to TORS. Patients were instructed not eat after midnight the day prior to the injection. The oropharynx was anesthetized using a topical formulation of 2% lidocaine solution (7 cc) and 2% lidocaine jelly (7 cc), combined to an artificial sweetener. The mixture was applied to the tonsillar fossa and lateral pharyngeal wall bilaterally using an angiocatheter. A videolaryngoscope or C-MAC laryngoscope were used to visualize the tumor in order to perform the injections. A total dose of 1 to 3 mCI of 110 MBq Tc99m sulfur colloid (unfiltered, TechneLite; Lantheus Medical Imaging, North Billerica, MA) in 1 cc syringe (volume of 0.4–0.6 mls) was injected. The injection was performed at four distinct quadrants at the visible edge of the tumor (medial, lateral, posterior and anterior). Approximately 0.1 cc were injected in each quadrant. The injections were performed using an angled laryngeal injection needle or spinal need. After injection of 99mTC-sulfur colloid, immediate and dynamic images (planar and single photon emission computed tomography (SPECT) /CT) were acquired using a Symbia T6/T16 (Siemens Healthineers, Erlangen, Germany) hybrid system. A flow study was obtained 2 min following the last injections. Delayed SPECT-CT images were subsequently acquired every 5 min for a total duration of 30 min.

### Statistical analysis

All variables were presented with medians and ranges for continuous data, and percentages for binary data. Time to event analysis was performed for recurrence and survival outcomes with time-to-event measured from the date of surgery to the date of contralateral nodal failure, locoregional failure (LRF), or date of death for overall survival (OS).

## Results

### Population

Thirteen patients underwent pre-operative SPECT-CT lymphatic mapping followed by TORS resection and ipsilateral neck dissection (Table 1). Neck dissection included levels 2–4. Lymphoscintigraphy was successful in 11/13 (84.6%) patients, showing adequate radiotracer migration to the nodal basins. The mean follow-up duration was 15.4 months (3–34). There was 7 (53.8%) female and 6 (46.2%) male patients. The median age at diagnosis was 61 years (42–74). The majority of patient (n = 10, 76.9%) had a known history of tobacco smoking (1.5–60 pack/year). Two (15.4%) patients were active smokers, 8 (61.5%) were previous smokers, and 3 (23.1%) were never smokers. Most patients have history of alcohol use (n = 10, 76.9%). Most patients (n = 10, 76.9%) had primary tumors located in the tonsillar fossa (n = 7 right, n = 3 left), while 3 (23.1%) patients had base of tongue tumors (n = 3 right, n = 0 left). Tumors were staged pT1 for 7 (53.8%) patients and pT2 for 6 (46.2%) patients. Six (46.2%) patients were staged pN0, 3 (23.1%) patients were pN1, 1 (7.7%) patient was pN2a for and 3 (23.1%) patients were N2b.

**Table 1:** Patient’s characteristics

Parameters	n (%)
Age (years)	
Range	42–74
Mean	60
Median	61
Gender	
Female	7 (53.8)
Male	6 (46.2)
Tumor site	
Tonsillar fossa	
Right	7 (53.8)
Left	3 (23.1)
Base of tongue	
Right	3 (23.1)
Left	0 (0.0)
Soft palate	0 (0.0)
TNM classification (AJCC 7th Edition)	
pT1	7 (53.8)
pT2	6 (46.2)
pT3	0 (0.0)
pT4	0 (0.0)
pN0	6 (46.1)
pN1	3 (23.1)
pN2a	1 (7.7)
pN2b	3(23.1)
pN2c	0 (0.0)
pN3a-b	0 (0.0)
cM0	13 (100.0)
cM1	0 (0.0)
Adjuvant radiotherapy	
Yes	4 (30.8)
No	9 (69.2)
Smoking status	
Current smoker	2 (15.4)
Ex-smoker	8 (61.5)
Non-smoker	3 (23.1)
Alcohol use	
Yes	10 (76.9)
No	3 (23.1)
Follow-up (months)	
Range	3–34
Mean	15.4
Median	14
Locoregional recurrence	
Primary site	1 (7.7)
Ipsilateral neck recurrence	0 (0.0)
Contralateral neck recurrence	0 (0.0)
Deaths	0 (0.0)

### Tumor description

Mean tumor size was 1.8 cm (0.9–3.2 cm). One (7.7%) patient had one positive mucosal margin on final pathology, although all frozen section margins were initially reported as negative. Six (46.2%) patients had close margins on final pathology. The majority of patients (n = 10, 76.9%) stained positive for p16 (> 70% on immunohistochemical staining), used as a surrogate for HPV status. The mean distance for tumors from midline was 0.837 cm (0.0–1.58 cm) Three (23.1%) patients showed evidence of perineural invasion, and 3 (23.1%) patients had evidence of lymphovascular invasion. (Table 2).

**Table 2:** Tumor description

Parameters	n (%)
Mean tumor size (mm)	18.1
Positive resection margin(s)	
Yes	1 (7.7)
No	12 (92.3)
Close resection margin(s) (< 5 mm)	
Yes	6 (46.2)
No	7 (53.8)
p16 status	
Positive	10 (76.9)
Negative	3 (23.1)
Distance from midline distance (cm)	
Median	1.05
Range	(0.0–1.58)
Perineural invasion	
Yes	4 (30.8)
No	9 (69.2)
Lymphovascular invasion	
Yes	3 (23.1)
No	10 (76.9)

### Lymphatics description

Lymphatic drainage patterns and nodal status are summarized in Table 3. Pre-operative lymphoscintigraphy showed ipsilateral nodal drainage for 10 (76.9%) patients, 1 (7.7%) patient had bilateral lymphatic drainage, 1 (7.7%) patient showed drainage to the ipsilateral retropharynx. Migration of the radiotracer was not visualized for 2 (15.4%) patients. No patients showed lymphatic drainage only to the contralateral neck. SPECT-CT labelled 1 lymph node for 3 (23.1%) patients, 2 lymph nodes for 6 (46.2%) patients, and 3 lymph nodes for 2 (15.4%) patients. No patients showed lymphatic drainage to more than 3 lymph nodes. Neck dissection identified a median of 27.0 (11–44) lymph nodes. Seven (53.8%) patients showed positive lymph nodes on final pathology; 4 (30.8%) patients had 1 positive LN, 3 (23.1%) patients had 2 positive LNs and no patients had > 2 positive LNs. One (7.7%) patient demonstrated pathological evidence of minor extra-nodal extension (< 0.2 cm). This patient did not receive adjuvant therapy due to post-operative complications.

**Table 3:** Lymphatic drainage according to SPECT-CT and neck dissection

Parameters	n (%)
Labelled LN distribution	
Ipsilateral-only	10 (76.9)
Contralateral-only	0 (0.0)
Bilateral	1 (7.7)
Retropharyngeal	
Ipsilateral	1 (7.7)
Contralateral	0 (0.0)
Not visualized	2 (15.4)
Number of labelled LN	
1	3 (23.1)
2	6 (46.2)
3	2 (23.1)
> 3	0 (0.0)
Number of removed LNs	
Range	11–44
Median	27.0
Pathologically involved LN	
Yes	7 (53.8)
No	6 (46.2)
Number of pathologically involved LN lymph nodes	
0	6 (46.2)
1	4 (30.8)
2	3 (23.0)
> 2	0 (0.0)
pENE	
Yes	1 (7.7)
No	12 (92.3)

LN, lymph node

### Adjuvant therapy

Nine patients (64.3%) had indications for PORT. Of these, 5 patients (38.5%) received PORT of 60 Gy in 30 fractions. Of those patients who had indications for radiation therapy (n = 9), but did not receive treatment (n = 4), the reasons were patient refusal in 1 patient (11%), unfit patient due to poor performance status in 1 patient (11.1%), and history of scleroderma/ connective tissue disease in 2 patients (22.2%). Among patients with close resection margin(s) (n = 6), 3 (50.0%) received PORT.

### Early oncological outcomes

Patients were followed for a median duration of 15.4 months (3–34 months). All patients were alive at the latest follow-up visit (Overall survival 100%). There were no observed cases (0.0%) of contralateral neck failure. One patient (7.7%) developed a locoregional failure at the primary site. This patient had an early local recurrence in the tonsillar fossa 5 months following primary treatment,. This patient had an initial pathology indicating negative margins, without adverse features and no nodal involvement. The patient received definitive concurrent chemoradiation therapy of 70 Gy in 35 fractions and 3 cycles of high dose cisplatin (100 mg/m^2^ every 3 weeks) for the recurrent disease. The patient has been followed for 18 months and shows no subsequent recurrence.

## Discussion

In the present study, we report early oncological outcomes of primary surgical treatment of lateralized early stage OPSCC. Our results demonstrate excellent overall survival outcomes, low rate of locoregional recurrence (7.7%), with no cases of contralateral neck failure. Many patients with lateralized tumors in the oropharynx treated with surgical resection undergo unilateral neck dissection in the absence of contralateral disease on axial imaging, notwithstanding the fact that occult nodal disease may occur in the contralateral neck with a low reported rate estimated to 7.4% of patients [6]. Here we demonstrate the feasibility of using lymphatic mapping with SPECT-CT to select patients for unilateral neck treatment. Our group has previously reported the feasibility of using this imaging modality to identify lymphatic drainage patterns, and now we demonstrate that treatment of the contralateral neck informed by lymphatic mapping provides acceptable early disease control in the contralateral neck and identifies patients at risk for contralateral nodal drainage [10].

In the present study, none of the patients subsequently failed in the contralateral neck. Several reports have consistently demonstrated low rates of contralateral neck failure for lateralized tumors [8, 9]. In a recent literature review, Al-Mamgani et al. [12] assessed regional recurrence for primary unilateral irradiation of oropharyngeal cancers. This review included 1116 patients treated for OPSCC and revealed an overall incidence of 2.4% for contralateral regional failure. In addition, the authors noted that midline tumor involvement was the most significant factor predictive for contralateral neck failure. Another recently published study in the surgical literature [13] showed in a cohort of 81 patients treated for T1-2 N0-2b squamous cell carcinomas of the tonsillar fossa, no cases of contralateral neck failure with an average follow-up of 5.7 years.

One patient in our study showed bilateral neck lymphatic drainage observed on pre-operative lymphoscintigraphy, with no concerning lymph nodes noted on CT and MRI imaging, and with a primary tumor involving the tonsillar fossa with soft palate invasion but not encroaching (> 1 cm) the midline. The patient was managed by primary site resection and unilateral neck dissection, given the absence of gross disease in the contralateral neck. Furthermore, the pre-operative imaging was suggestive of more than one ipsilateral lymph node and the initial plan was for planned adjuvant radiotherapy as the patient had declined upfront chemoradiotherapy due to concerns of toxicity related to systemic therapy. As such, the patient received adjuvant therapy to manage the potential for occult nodal metastases to the contralateral neck. Other potential treatment options exist for management of contralateral lymphatic drainage in the absence of gross disease. De Veij Mestdagh et al. are evaluating the use of sentinel lymph node biopsy for identification of occult contralateral neck disease in patients with lateralized oropharyngeal cancers [14]. The benefit of this approach is that pathologic disease can be identified and properly staged. Alternatively, one may consider contralateral neck dissection to identify pathologically occult nodal disease. However, the drawback of either of these approaches is the potential morbidity of further surgical intervention in the contralateral neck, despite the fact the patient was likely and did receive adjuvant radiotherapy to the undissected contralateral neck. Regardless of the approach, we would advocate for some management of the contralateral neck in patients with lymphatic drainage to that side because of the possibility of occult nodal disease.

A lymphatic mapping approach also identified unpredictable patterns of lymphatic drainage such as drainage to retropharyngeal lymph nodes. Drainage to the retropharyngeal lymph node basin may be relatively uncommon with reports describing a prevalence of less than 10% [15–17], and most commonly associated with pharyngeal wall primary tumors. In patients treated with a primary surgical modality, dissection of retropharyngeal nodal basin is not commonly performed although some centres have published techniques to dissect the retropharyngeal nodal basin [18, 19] with either a transcervical or transoral approach. Use of lymphatic mapping may help identify patients at risk for retropharyngeal nodal drainage. In the present series, we demonstrated one patient who had evidence of retropharyngeal lymphatic drainage. This same patient had an HPV negative primary tumor of the tongue base and glossotonsillar sulcus with a single metastatic lymph node with minor extranodal extension and with no evidence of pathologic disease in the dissected retropharyngeal basin. In this case, the lymphatic mapping study helped identify a potential route of spread requiring surgical management, albeit there was no evidence of pathologic disease in this basin. This finding may suggest that lymphatic mapping may further add value in directing treatment of patients with oropharyngeal cancers.

Our results also support a promising role for treatment deintensification among patients with lateralized oropharyngeal tumors. A cautious selection of patients and avoidance of unnecessary treatment to the contralateral neck, can significantly reduce short- and long-term treatment related toxicities [20]. A personalized management of patients can potentially reduce costs associated to treatment related toxicities, such as hospital admission (pneumonia, dysphagia, malnutrition, etc.) and additional interventions (gastrostomy feeding tube placement, tracheostomy, etc.) [21, 22]. Jensen et al. [23] has showed that incapacitating side effect of radiation therapy can be significantly reduced with ipsilateral treatment of the neck. Patients with unilateral treatment showed significantly lower levels of xerostomia, dysphagia, hoarseness, fibrosis and edema in comparison to patients that underwent bilateral irradiation. Other potential benefit includes improvement of quality of life and level of functioning [24]. Several ongoing prospective randomized trials are currently assessing the role of deintensification therapy for OPSCC [25].

Limitations of this study include a limited number of patients, the retrospective nature of the data, and a conservative long-term follow-up duration. Approximately three quarters of patients (76.6%) in our study showed positive staining for p16, demonstrating heterogenicity of the oropharyngeal tumors treated, however, previous studies did not show significant differences for nodal metastasis between HPV positive and negative OPSCC [26]. In addition, although SPECT-CT lymphoscintigraphy appears as an interesting tool for the assessment of oropharyngeal malignancies, it should be used with caution for tumors close to midline (soft palate, tongue base) which may not be amenable to ipsilateral treatment due to an increased risk of bilateral lymphatic drainage [12].

## Conclusion

The use of pre-operative SPECT-CT lymphoscintigraphy prior to unilateral primary surgical management of early stage OPSCC is an accurate and reliable tool which allows us to predict lymphatic drainage of tumors without compromising oncological outcomes. The inclusion of lymphatic mapping in the initial management algorithm of OPSCC can improve characterization of lymphatic drainage to the contralateral neck and offer a personalized treatment based on tumor features.

## Data Availability

The datasets used and/or analysed during the current study are available from the corresponding author on reasonable request.
